# Characterizing the bacterial microbiota in different gastrointestinal tract segments of the Bactrian camel

**DOI:** 10.1038/s41598-017-18298-7

**Published:** 2018-01-12

**Authors:** Jing He, Li Yi, Le Hai, Liang Ming, Wanting Gao, Rimutu Ji

**Affiliations:** 1 Key Laboratory of Dairy Biotechnology and Bioengineering, Ministry of Education, College of Food Science and Engineering, Inner Mongolia Agricultural University, Hohhot, Inner Mongolia China; 2 Camel Research Institute of Inner Mongolia, Alxa, Inner Mongolia China

## Abstract

The bacterial community plays important roles in the gastrointestinal tracts (GITs) of animals. However, our understanding of the microbial communities in the GIT of Bactrian camels remains limited. Here, we describe the bacterial communities from eight different GIT segments (rumen, reticulum, abomasum, duodenum, ileum, jejunum, caecum, colon) and faeces determined from 11 Bactrian camels using 16S rRNA gene amplicon sequencing. Twenty-seven bacterial phyla were found in the GIT, with Firmicutes, Verrucomicrobia and Bacteroidetes predominating. However, there were significant differences in microbial community composition between segments of the GIT. In particular, a greater proportion of *Akkermansia* and Unclassified Ruminococcaceae were found in the large intestine and faecal samples, while more Unclassified Clostridiales and Unclassified Bacteroidales were present in the in forestomach and small intestine. Comparative analysis of the microbiota from different GIT segments revealed that the microbial profile in the large intestine was like that in faeces. We also predicted the metagenomic profiles for the different GIT regions. In forestomach, there was enrichment associated with replication and repair and amino acid metabolism, while carbohydrate metabolism was enriched in the large intestine and faeces. These results provide profound insights into the GIT microbiota of Bactrian camels.

## Introduction

Gut microbes of mammals are now regarded as having important roles in the maintenance of health and modulation of disease. Recent advances in microbial ecology have shown that the balance of the gastrointestinal (GI) microbial community is critical to maintenance of host health. Perturbation of this microbial composition is closely related to diseases including diabetes^1^, obesity^2^, inflammatory bowel disease^3^ and cancer^4,5^. However, most studies have investigated the characteristics of the gut microbial community in faeces. Microbiotic profiling of the gastrointestinal tract (GIT) itself can only be found in a few short studies, for example of pig^6^, house mouse^7^, dairy cow^8^, horse^9^, rat^10^, broiler chicken^11^ and dog^12^.

The Bactrian camel is a very hardy animal which can live in deserts or semi-deserts. It can adapt to the harsh environments, such as arid, poor grazing, hot and cold. Camels are a means of conveyance and producers of milk, meat and fur. Research has shown that Bactrian camels are an ideal model for describing desert adaptations because of their ability to tolerate harsh desert ecological conditions^13,14^. Bactrian camels have ability to adapt to low quality diet. It can eat salt-tolerant vegetation such as *Chenopodiaceae*, *Compositae and Leguminosae* plants. they also have capacity to ingest virtually any kind of vegetation including shrubs and trees^15^.

The digestive systems of camels are different from cattle and sheep, in contrast to the four chambered stomach of most true ruminants, the Bactrian camel stomachs have only three chambers with no omasum^16^. In addition, camels retain feed particles in forestomach for much longer than other large herbivores^17,18^. Moreover, previous study has found that in Camelus dromedarius large particles have slightly longer retention time than small particles in forestomach. But, retention times of fluid, large and small particles are similar in the intestine^19^. Characterization of the Bactrian camel microbiota is therefore important. Recently, the microbiota in camel rumen and faeces have been detected^20–22^. However, microorganisms elsewhere in the GIT have received little attention. In this study, we undertook bacterial 16S rRNA gene sequence-based profiling of the Bactrian camel GIT. We describe the characteristics of microbiota in different parts of the GIT and identify similarities with faecal microbes.

## Materials and Methods

### Animals and sample collection

Eleven adult Bactrian camels were used in this study. All Bactrian camels were distributed in the Inner Mongolia XilinGol League, China. They mainly eat Chenopodiaceae, Compositae and Leguminosae plants such as *Agriophyllum pungens*, *Ceratoides latens*, and *Nitraria tangutorum*. All 11 camels were slaughtered, and samples were taken from eight segments, including the rumen (LW), reticulum (WW), abomasum (ZW), duodenum (12Z), jejunum (KC), ileum (HC), caecum (MC), colon (JC), and from faeces (FB). In total, 99 samples were collected and frozen rapidly in liquid nitrogen, then stored at −80 °C until DNA extraction. The experiment was conducted according to the animal ethics guidelines of the Key Laboratory of Dairy Biotechnology and Bioengineering, and approved by the Animal Ethics Committee of Inner Mongolia Agricultural University.

### 16S rRNA sequencing

Microbial genomic DNA was extracted from samples using a Mag-Bind Soil Kit (Omega, M5635). DNA quality was determined with a NanoDrop spectrophotometer and by 0.8% agarose gel electrophoresis. The V4 hypervariable region of bacterial 16S rRNA genes was amplified by PCR with initial denaturation at 98 °C for 2 min; 25–30 cycles of denaturation at 98 °C for 15 s, annealing at 55 °C for 30 s, and extension at 72 °C for 30 s; and a final extension at 72 °C for 5 min. PCR products were purified from 2% agarose gels, and quantitated using the Quant-iTTMPicoGreen® dsDNA Assay Kit (Life Technologies, Grand Island, NY, USA). Libraries were prepared using a TruSeq Nano DNA LT Kit (Illumina). Purified amplicons were sequenced using an Illumina MiSeq platform at Personal Biotechnology Co., Ltd., Shanghai, China.

### Bioinformatics analyses

We used Greengenes 13.8 to classify taxonomic abundance^23^. Bacterial operational taxonomic units (OTUs) were generated using the UCLUST function in Quantitative Insights into Microbial Ecology (QIIME, v.1.8.0)^24^. Alpha diversity of Chao1, the Shannon index and phylogenetic diversity were calculated by QIIME. Principal coordinate analysis (PCoA) was conducted using the weighted UniFrac distance method^25^. A hierarchical clustered heatmap was used to reveal the relative abundance of genera in each sample^26^. Differences in the overall bacterial composition of the eight gastrointestinal tract segments plus faeces were tested via correlation using Microsoft Excel. We used PICRUSt to predict the functional gene content of microorganisms^27^. The predicted function are precalculated for genes in KEGG database^28^. The differences between faeces and other segments were compared by STAMP^29^.

### Real-time PCR (RT-PCR) analysis of the total number of bacteria

RT-PCR was used to determine 16S rRNA gene copy numbers in GIT bacteria with universal bacterial primers 1114 F (5′-CCATTGTAGCACGTGTGTAGCC-3′) and 1221 R (5′-CGGCAACGAGCGCAACCC-3′), in samples from all nine sampling sites^25^. The PCR reactions were performed in an ABI Step One real-time PCR machine (Applied Biosystems, Foster City, CA) with denaturation at 95 °C for 5 min, and 40 cycles of 15 s at 95 °C and 60 °C for 30 s.

### Statistical analysis

Differences in alpha diversity, relative abundance of taxa, and concentrations of bacterial populations among different groups were analysed using the Kruskal-Wallis rank sum test in R. The Kruskal-Wallis rank sum test with Dunn test and Benjamini-Hochberg correction were chosen for multiple comparisons of groups. ADONIS was used with 999 permutations in QIIME to quantify the effect size of variables explaining weighted UniFrac distances. All *p* values from the Kruskal-Walis H-test and Welch’s t-test of the KEGG pathways were corrected for an FDR using the Benjamini-Hochberg method.

### Data availability

The sequencing data from this study were deposited in the NCBI Sequence Read Archive (SRA) under accession no. SRP114499.

## Results

### Diversity of the bacterial community in the Bactrian camel GIT

**Figure 1:**
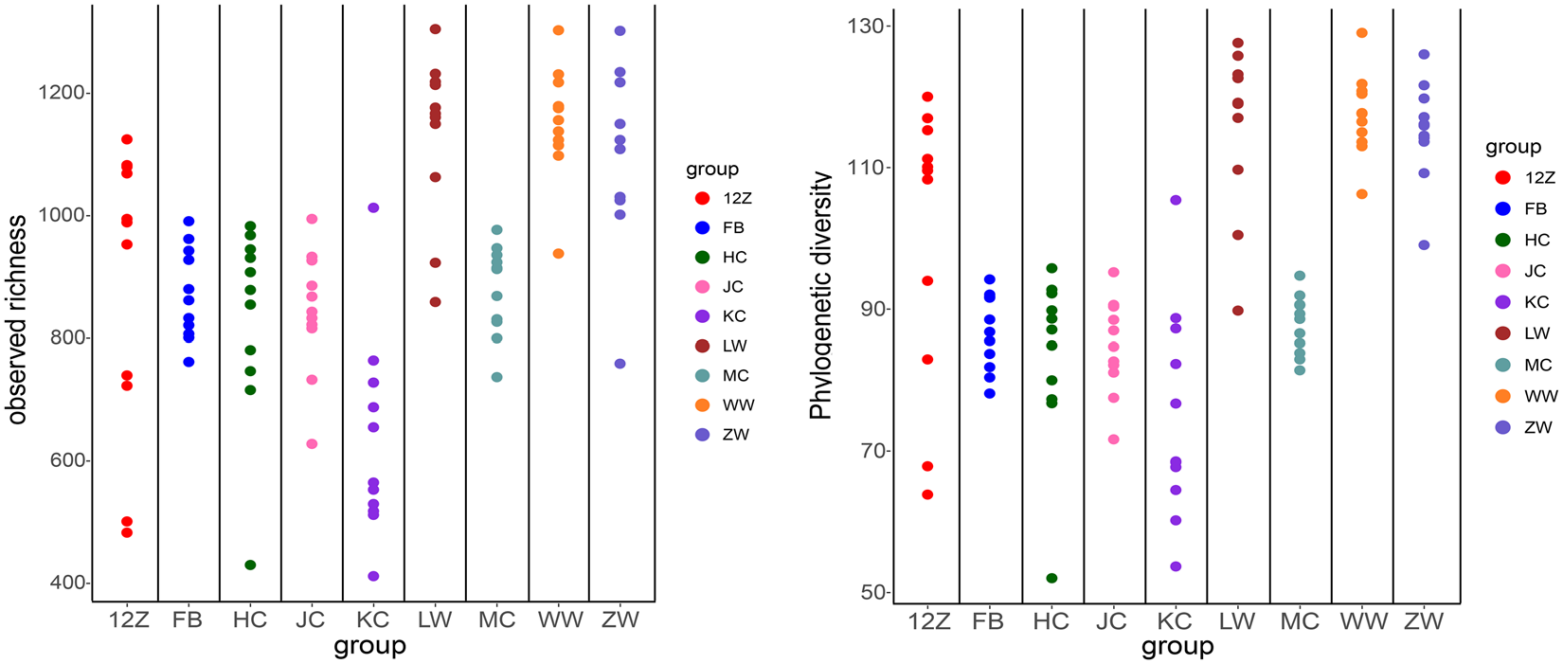
Diversity measurements. (**a**) Observed richness and (**b**) phylogenetic diversity measurements at each GIT site for 11 Bactrian camels. 12Z: duodenum samples; FB: faeces; HC: ileum; JC: colon; KC: jejunum; LW: rumen; MC: caecum; WW: reticulum; ZW: abomasum samples.

GIT microbiota were analysed based on 99 sequenced samples (nine sites from each of 11 Bactrian camels), which generated 4,079,128 valid reads. Each sample was covered by an average of 36,315 sequences; 4,035 OTUs were detected, assigned based on 97% nucleotide sequence identity between reads. Individually based rarefaction curves were generated to assess whether sampling was sufficient for each segment of the GIT (Fig. S1). The Good’s coverage ranged from 98% to 99% for all animals (Table S1). The observed richness and phylogenetic diversity were used to evaluate the community diversity of each sample (Fig. 1).

The number of OTUs, Shannon diversity index (*p* < 0.05) and Chao1 value (*p* < 0.05) differed significantly among tissues (Table 1). Notably, the Shannon diversity index and Chao1 value in the rumen reticulum were significantly higher than those in the other sites (Table 1), as well as richness and diversity were lowest in jejunum samples. Furthermore, marked inter-camel variations were observed in community diversity levels (Table S1).

**Table 1:** Sequencing information and significance analysis alpha diversity.

Region	Valid reads	OTUs	Chao1 value	Shannon index
rumen	39315	1560.27	1144.21^c^	8.00 ^c^
reticulum	40765	1566.73	1156.33^c^	8.19^c^
abomasum	39449	1514.18	1105.27^c^	7.58^bc^
duodenum	37097	1241.91	889.44^a^	6.71^ab^
jejunum	37879	949.91	641.18^b^	5.82^a^
ileum	40061	1232.36	848.59^ab^	6.34^a^
caecum	45321	1348.55	890.39^ab^	6.60^ab^
colon	45782	1316.18	853.67^ab^	6.47^a^
faeces	45160	1325.82	875.40^ab^	6.49^a^

Note: Means in the same column with different superscript letters denote significant differences between each group.

**Figure 2:**
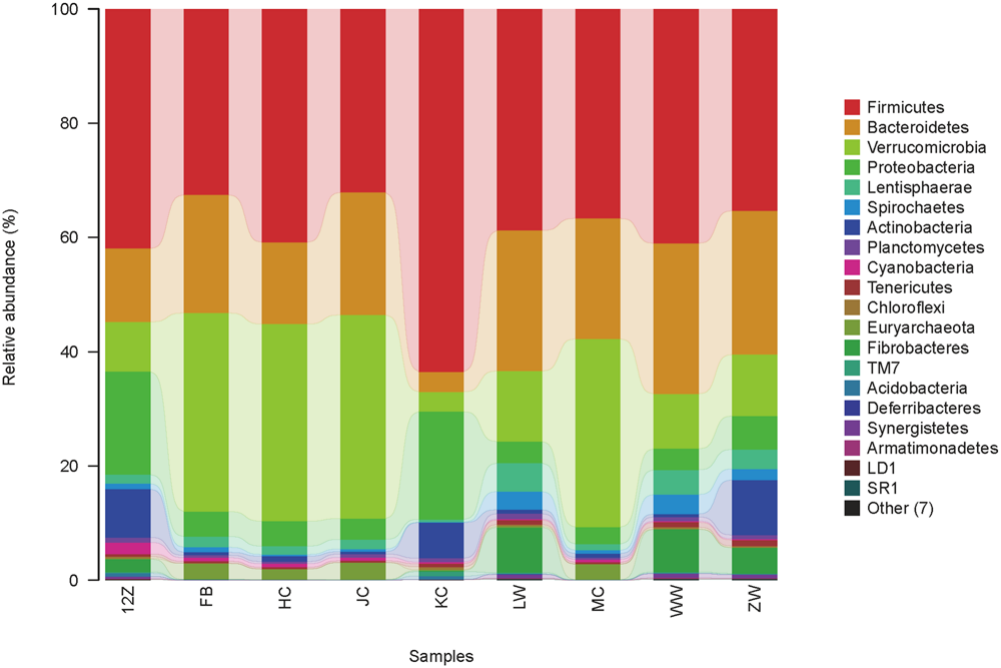
Relative abundances of sequences belonging to different phyla in the Bactrian camel gastrointestinal tract (GIT). 12Z: duodenum samples; FB: faeces; HC: ileum; JC: colon; KC: jejunum; LW: rumen; MC: caecum; WW: reticulum; ZW: abomasum samples.

Twenty-seven bacterial phyla were identified in the Bactrian camel GIT (Fig. 2, Table S2). The taxa principally belonged to Firmicutes (39.97%), Verrucomicrobia (21.10%) and Bacteroidetes (18.94%). Firmicutes were dominant in all bacterial communities along the GIT. Bacteroidetes was the second most dominant phylum in the forestomach, while Verrucomicrobia was the second most abundant in the ileum, caecum, colon and faeces (Table S3). Only Firmicutes, Verrucomicrobia, Bacteroidetes, Planctomycetes, Proteobacteria, Spirochaetes, Tenericutes, Actinobacteria, Cyanobacteria and Lentisphaerae were found in all samples. The duodenum harboured the most phyla (27 phyla), while the lowest number of phyla (19 phyla) was observed in the ileum.

**Figure 3:**
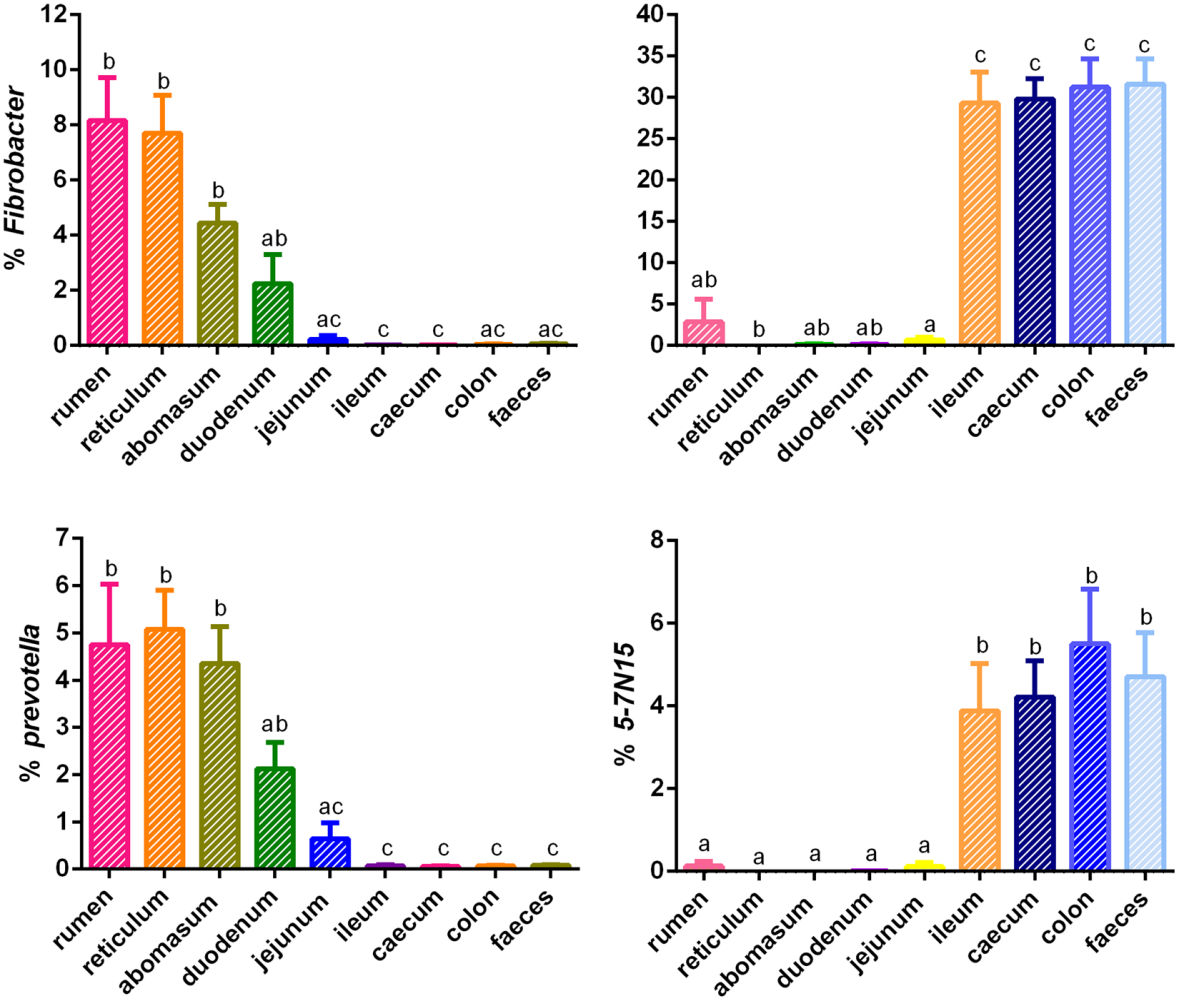
Richness of particular genera in the Bactrian camel gut. Data show means ± SEM. Bars within a chart marked with different ower-case letters are significantly different (*P* < 0.05).

Considering genera, 282 were detected in the Bactrian camel GITs (Table S4). however, 49.13% of all sequences were not identified. The most prevalent genera in the GITs included *Akkermansia*, *Fibrobacter*, *Prevotella*, *5–7N15*, *Pseudomonas*, *Burkholderia*, and *Lactobacillus*, as well as unclassified genera belonging to the families Christensenellaceae, Ruminococcaceae, Bifidobacteriaceae, f_RFP12 and BS11, and the orders Clostridiales and Bacteroidales (Fig. S3 and Table S5). Dominant taxa such as *Prevotella*, *Fibrobacter*, unclassified Bacteroidales, unclassified BS11 and unclassified Clostridiales were enriched significantly in forestomach sites (Fig. 3 and Table S5). However, *Akkermansia*, *5–7N15* and unclassified Ruminococcaceae were enriched more in the large intestine and ileum than in the other GIT samples (Fig. 3 and Table S5). Other phyla such as *Lactobacillus*, *Burkholderia* and *Pseudomonas* were more abundant in the duodenum and jejunum. The relative abundance of unclassified Bifidobacteriaceae was remarkably higher in the abomasum, duodenum and jejunum than elsewhere in the GIT (Table S5).

### Characterization of microbiota along the Bactrian camel GIT

**Figure 4:**
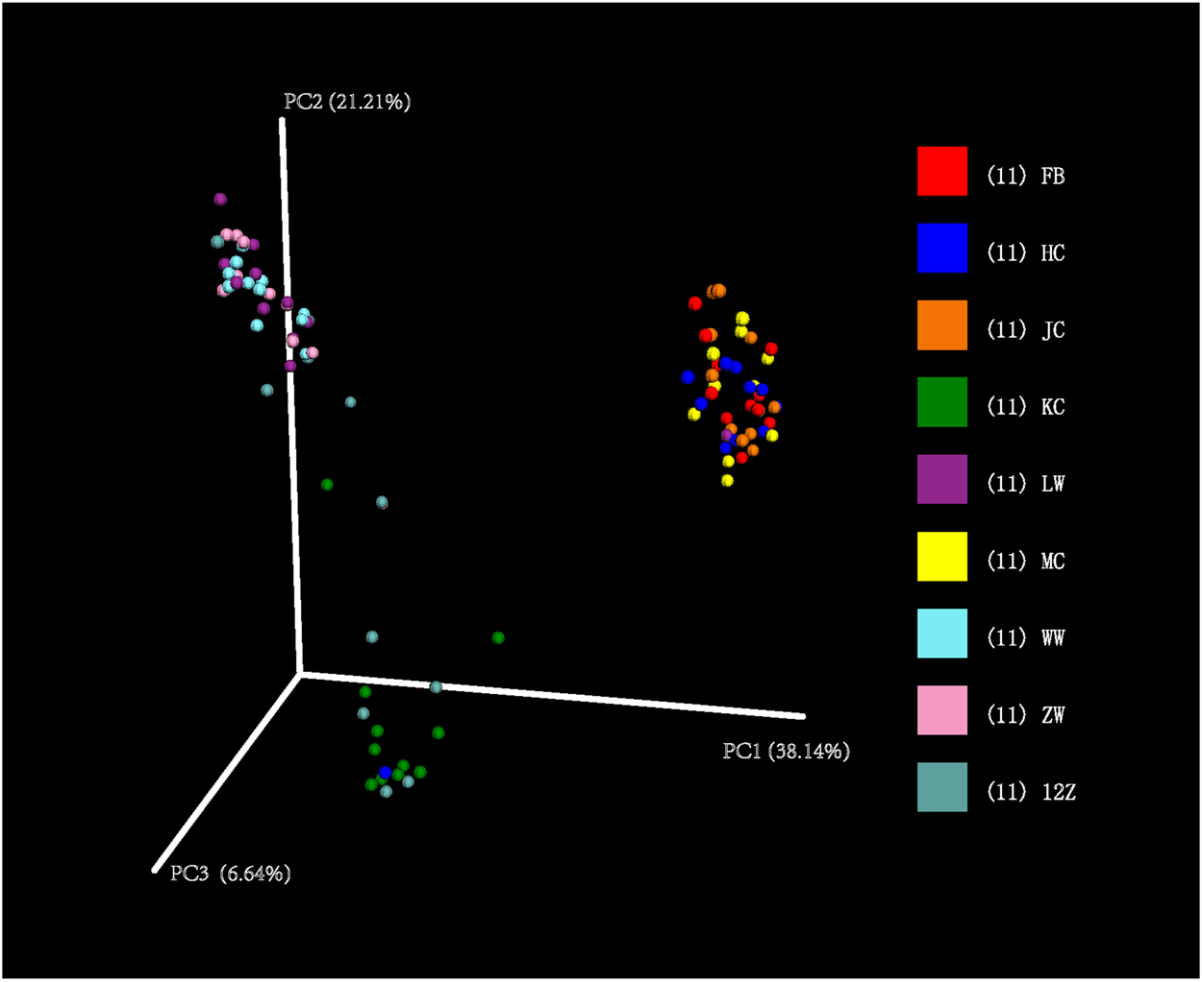
Principal coordinate analysis of microbial community membership (weighted UniFrac distance). 12Z: duodenum samples; FB: faeces; HC: ileum; JC: colon; KC: jejunum; LW: rumen; MC: caecum; WW: reticulum; ZW: abomasum samples.

The differences in microbial communities between different parts of the GIT were measured by unweighted and weighted UniFrac beta diversity measures based on ADONIS at the genus level. This showed many differences between GIT origins, at *P* = 0.001 (ADONIS, R^2^ = 0.57) using weighted UniFrac distance measures. This was further supported by PCoA of weighted UniFrac distances, which showed the difference in distribution of microbes among sites, and that the bacterial communities of (i) the forestomach (rumen, reticulum, abomasum), and (ii) the ileum and large intestine (caecum, colon and faeces) were spatially separated from each other (Fig. 4). Similarly, Ward’s cluster heatmap results supported the PCoA findings and indicated the high or low-prevalence genera in each sample (Fig. S3).

To determine the association among the microbiota in different sites of the GI tract, correlation analysis was conducted. Table 2 shows that the composition of the microbial communities differed significantly between regions of the upper and lower GIT. The microbiota of the rumen, abomasum and reticulum had high similarity. High similarities were also observed between each region of the lower GIT (caecum, colon and faeces). There was also high similarity between the ileum and the lower GIT.

**Table 2:** Correlations of genus abundance among different GI tract segments.

	reticulum	abomasum	duodenum	jejunum	ileum	caecum	colon	faeces
rumen	0.9895	0.9071	0.7831	0.6790	0.4934	0.4484	0.4255	0.4387
reticulum		0.9165	0.7882	0.6700	0.3939	0.3515	0.3271	0.3416
abomasum			0.8862	0.6780	0.3748	0.3361	0.3131	0.3264
duodenum				0.8568	0.3867	0.3269	0.2983	0.3109
jejunum					0.3839	0.2873	0.2445	0.2555
ileum						0.9896	0.9839	0.9846
caecum							0.9966	0.9964
colon								0.9992

Note: eleven samples were used to calculate correlation for each group.

### Total bacterial populations

**Figure 5:**
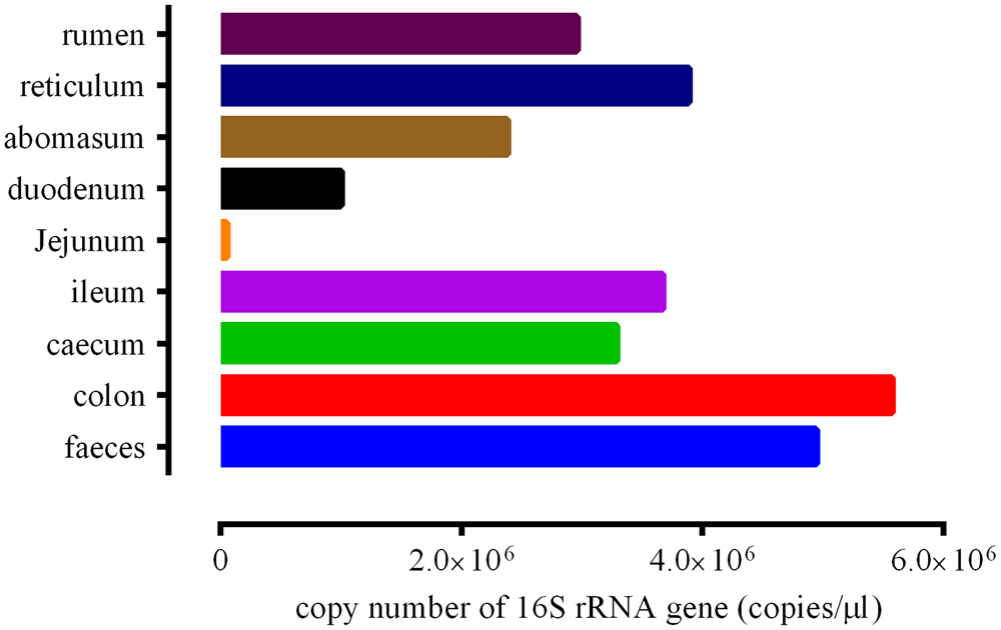
Bacterial density through the GIT of Bactrian camel (data are means).

Total bacterial populations in various GIT anatomical sites were assessed by RT-PCR to measure the total copy number of bacterial 16S rRNA genes (Fig. 5). The density of bacteria in different GIT segments of the Bactrian camel is remarkably distinct. Higher bacterial counts were observed in the colon and faeces. The populations of bacteria in the jejunum were significantly lower than in the other sites. Moreover, the bacterial population in forestomach were also lower than the faeces.

### Predicted functions of microbiota

**Figure 6:**
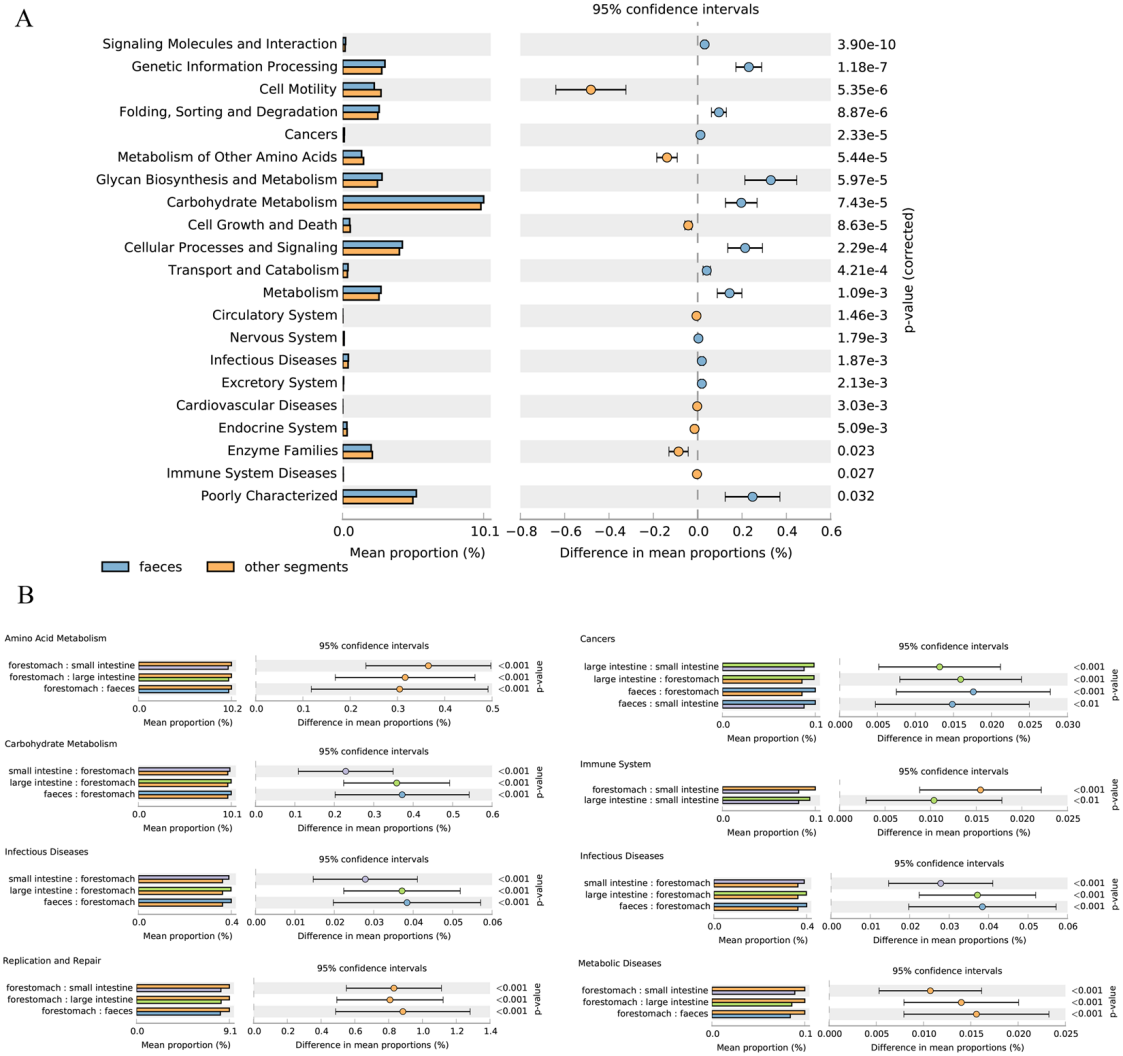
Microbial functional predictions. 12Z: duodenum samples; FB: faeces; HC: ileum; JC: colon; KC: jejunum; LW: rumen; MC: caecum; WW: reticulum; ZW: abomasum samples. (**A**) Comparison of functional pathway between microbes of feces and other contents. (**B**) Comparison of microbial functions associated with metabolism and disease among forestomach, small intestine, large intestine and faeces.

To investigate microbiotic functions in the samples, we performed functional analysis using PICRUSt (Fig. 6). Forty-one gene families were identified in all samples, of these, many of the genes function in membrane transport, carbohydrate metabolism, amino acid metabolism, replication and repair, and energy metabolism. The prevalence of the 40 specific gene families was remarkably different among GIT sites (Table S6). In the forestomach, the relative abundances of genes involved in amino metabolism, replication and repair were significantly higher than was observed in the other anatomical sites. However, the bacteria in the lower GIT were significantly enriched in categories which associated with carbohydrate metabolism (Fig. 6A and Table S6). Comparing predicted KEGG function between faeces and other segments, we detected that they had significant enrichment in the predicted functions of microbiota (Fig. 6A). In particular, by comparing the functions associated with disease in different sites, we noticed that the forestomach contained more microbial functions than other segments for metabolic disease and immune system. But, the functions in cancer and infectious disease were detected high proportion in gut tracts especially in duodenum and jejunum (Fig. 6B and Table S6).

## Discussion

Complex GIT microbial communities are believed to provide benefits to their host^30^, and are receiving increasing attention. However, the characteristics and distribution of the microbial community in the Bactrian camel GIT remains unclear. Here, we performed second generation sequencing to investigate these issues. We found significant differences in microbial community composition between different segments of the GIT in Bactrian camel. Analysis of the forestomach revealed greater relative abundances of Firmicutes and Bacteroidetes, whereas Firmicutes and Proteobacteria showed the highest relative abundances in the duodenum and jejunum. Firmicutes and Verrucomicrobia were the most abundant taxa in the ileum and large intestine (Fig. 2). The taxonomic groups represented within the Bactrian camel GITs were similar to those previously observed in the gastric environment and faeces of camels^20–22,31^. The different distribution of intestinal microbes was affected by the multiple factors such as species, individuals and diets^32^.

Interestingly, the dominant taxa belonging to Firmicutes in the Bactrian camel forestomach were unclassified Clostridiales, which were found in the rumen and became the main polysaccharide degraders in cattle^33^. However, unclassified Ruminococcaceae, which are commonly observed in the rumen and implicated in the degradation of starch and fibre in ruminant animals^34^, were enriched in the ileum and large intestine of Bactrian camels (Fig. S2). It is possible that these communities may contribute to further feed fermentation in the camel rumen^35^. Because camels have unique ability to survive on salt-tolerant vegetation and to digest poorly-digestible forage^15^, whereas such plants are not eaten by other herbivores like cattle, sheep and horses.

At the phylum level, Verrucomicrobia were significantly less abundant in the microbiota of the forestomach compared with the ileum and large intestine. The genus *Akkermansia* (phylum Verrucomicrobia) reached up to 31.64% of the total reads in some samples (Table S5). Previous research showed that *Akkermansia* in the intestinal tract may reduce obesity, diabetes, and inflammation in mice and humans^36,37^. *Akkermansia* may also play important roles in Bactrian camel biology. It is tempting to speculate that *Akkermansia* could harbour the necessary functions for Bactrian camels to deal with high blood glucose levels and tolerate a high dietary intake of salt, given that they do not develop diabetes or hypertension^13^. *Bifidobacteria* and *Lactobacillus* are considered probiotic microorganisms and are beneficial to the immunity of the host^38^. Probiotics may prevent pathogens from proliferating in the intestinal tract^39^. Previous researches have also been reported that *Bifidobacteria* can contribute to the gut health by production of inhibitory substances^40,41^. Here, the abundance of Unclassified Bifidobacteriaceae reached up to 9.01% of the total reads in the Bactrian camel abomasum. It maybe prevention of gastrointestinal diseases in the Bactrian camel. But, the high abundance of unclassifed Bifidobacteriaceae in the abomasum of the Bactrian camel is not entirely clear, and future studies are needed to clarify this issue.

Segmented distribution of gut microbes has already been reported in cow, mouse and pig^6,8,32,35,42^. In this study, different microbial communities were found between the forestomach, small intestine and large intestine; the distribution of gut microbes in ileum samples was closest to that in the large intestine (Fig. 4). Different anatomic regions in GI tracts have distinct physicochemical conditions such as intestinal flow rate, redox potential, oxygen concentration and availability of nutrients^43–45^. However, further study is required to explain the similarity between the ileal and large intestinal microbial communities observed here. The present study also confirmed that there is a decrease in bacterial diversity through the GIT to the jejunum where the lowest diversity was observed; after the jejunum, the microbial diversity increased again (Table 1 and Fig. 5). It was possibly due to the involvement of some bacteria in the degradation of food biomass that bypasses the forestomach and small intestine^32^.

Microbial functional analysis showed that the categories of amino acid metabolism, carbohydrate metabolism, replication and repair and membrane transport were the most abundant in our study, in agreement with previous studies on humans^46^, cattle^8^, porcine^6^ and mice^47,48^. The present study also revealed significant differences in bacterial function among different anatomical sites of the Bactrian GIT. For example, genes involved in replication and repair and amino acid metabolism were at their highest in forestomach samples. But genes relating to carbohydrate metabolism were more abundant in the hindgut. The reason for this phenomenon was that like non-ruminant such as porcine^6^, the forestomach and small intestine is related to digestion and absorption, while the large intestine is mainly responsible for microbial fermentation. Our study also found that the duodenum and jejunum contained higher microbiota which related with infection and cancer disease, this result suggested that the pathogen invasion may enrich in this section. In addition, the forestomach contained more microbial function than other segments for metabolic disease. it was associated with the retention of feed particles in camel rumen much longer than other large herbivores which can lead to a lower metabolic metabolism and food intake of camelid than ruminants^49^.

In conclusion, this study is the first to describe the characteristics of the microbial communities in the GIT of Bactrian camels. Changes in the composition and diversity of microbial communities were diverse in the different sites of the GIT. Though faecal microbiomes have remarkable similarity with those in the large intestine, faeces cannot fully represent the microbial profiles of GITs. Therefore, this study has some limitations, including the relatively small sample size and an inability to control for potentially important variables such as sex and age. In addition, the structures of microbial communities are influenced by the individual animal sampled. Further research is required to understand specific factors affecting the microbial community composition among gut segments and among individuals. There is another limitation is that because we only used the 16S rRNA hypervariable region V4 of bacteria for analysis, most sequences could only be annotated to the genus or family level. Whole-genome shotgun sequencing should be carried out to gain further insight into the GI microbial community in Bactrian camels.

## Electronic supplementary material

Supplementary Table S2 

Supplementary Table S4 

Supplementary information
