# Supplementary material for: Characterizing the bacterial microbiota in different gastrointestinal tract segments of the Bactrian camel
Source: Sci Rep. 2018 Jan 12;8:654. doi: 10.1038/s41598-017-18298-7 (PMC5766590; doi:10.1038/s41598-017-18298-7)

**Supplementary Figures and Tables**

**Characterizing** **the bacterial microbiota in different** **gastrointestinal tract segments of** **Bactrian camel**

Jing He^1^, Li Yi^1^, Le Hai^1^, Liang Ming^1^, Wanting Gao^1^, Rimutu Ji^1,2^

^1^Key Laboratory of Dairy Biotechnology and Bioengineering, Ministry of Education, College of Food Science and Engineering, Inner Mongolia Agricultural University, Hohhot, Inner Mongolia, China

^2^Camel Research Institute of Inner Mongolia, Alxa, Inner Mongolia, China

**Supplementary Table S1** Number of sequences, estimated sample coverage, diversity and operational taxonomic unit (OTU) richness in each sample

**Supplementary Table S2** The 16S rRNA copy number adjusted counts for phyla present in each sample. See Supplemental_Table_S1.csv.

**Supplementary Table S3** Comparison of the predominant phyla (average relative abundance ≥5% in at least one GIT region) in all samples across the gastrointestinal tract within Bactrian camels using the Kruskal-Wallis test. Means in the same row with different superscripts represent a significant difference.

**Supplementary Table S4** The 16S rRNA copy number adjusted counts for genera present in each sample. See Supplemental_Table_S2.csv.

**Supplementary Table S5** Comparison of the predominant genera (average relative abundance ≥5% in at least one GIT region) in samples across the gastrointestinal tract of Bactrian camels using the Kruskal-Wallis test. Means in the same row with different superscripts represent a significant difference (*P* < 0.05).

**Supplementary Table S6** Predicted functions of the bacterial microbiota throughout the GIT of Bactrian camels using the Kruskal-Wallis test. Means with the same superscript within the same row are significantly different at *P* < 0.05.

**Supplementary Fig S1** Summary of rarefaction results based on operational taxonomic units (OTUs) for each sample. 12Z: duodenum samples; FB: faeces samples; HC: ileum samples; JC: colon samples; KC: jejunum samples; LW: rumen samples; MC: caecum samples; WW: reticulum samples; ZW: abomasum samples.

**Supplementary Fig S2** Heatmap of hierarchy cluster results for the abundance of genera in different GIT segments. 12Z: duodenum samples; FB: faeces samples; HC: ileum samples; JC: colon samples; KC: jejunum samples; LW: rumen samples; MC: caecum samples; WW: reticulum samples; ZW: abomasum samples.

**Supplementary Tables**

**Table S1. Number of sequences, estimated sample coverage, diversity and operational taxonomic unit (OTU) richness in each sample**

| Region | Sample ID | Valid sequences | OTUs | Chao1 | Shannon | Coverage |
| --- | --- | --- | --- | --- | --- | --- |
| Rumen | LW4 | 31299 | 1159 | 859.00 | 7.8 | 0.9904 |
|  | LW5 | 37998 | 1506 | 1158.00 | 8.08 | 0.9909 |
|  | LW6 | 44553 | 1774 | 1177.33 | 8.44 | 0.9903 |
|  | LW7 | 39641 | 1713 | 1250.43 | 8.44 | 0.9897 |
|  | LW8 | 40657 | 1623 | 1215.50 | 8.4 | 0.9914 |
|  | LW9 | 33798 | 1666 | 1219.00 | 8.38 | 0.9867 |
|  | LW10 | 42198 | 1392 | 955.25 | 6.83 | 0.9918 |
|  | LW11 | 42409 | 1586 | 1225.00 | 7.66 | 0.9909 |
|  | LW12 | 41472 | 1569 | 1169.00 | 8.04 | 0.9911 |
|  | LW13 | 39376 | 1690 | 1298.50 | 8.38 | 0.9905 |
|  | LW14 | 39067 | 1485 | 1059.25 | 7.55 | 0.9897 |
| Reticulum | WW4 | 46254 | 1344 | 942.20 | 7.72 | 0.9939 |
|  | WW5 | 38983 | 1483 | 1093.00 | 7.93 | 0.9903 |
|  | WW6 | 36033 | 1481 | 1138.00 | 8.44 | 0.9905 |
|  | WW7 | 51557 | 1796 | 1173.20 | 8.23 | 0.9921 |
|  | WW8 | 37305 | 1691 | 1300.00 | 8.65 | 0.9896 |
|  | WW9 | 34248 | 1582 | 1218.00 | 8.31 | 0.9893 |
|  | WW10 | 41385 | 1481 | 1135.00 | 8.23 | 0.9923 |
|  | WW11 | 38369 | 1512 | 1117.00 | 7.76 | 0.9897 |
|  | WW12 | 39524 | 1554 | 1173.00 | 8.15 | 0.9908 |
|  | WW13 | 42517 | 1650 | 1182.50 | 8.32 | 0.9915 |
|  | WW14 | 42242 | 1660 | 1247.75 | 8.32 | 0.9912 |
| abomasum | ZW4 | 40369 | 1478 | 1036.00 | 8.08 | 0.9905 |
|  | ZW5 | 38129 | 1502 | 1007.00 | 6.2 | 0.9880 |
|  | ZW6 | 41430 | 1422 | 1046.25 | 7.76 | 0.9919 |
|  | ZW7 | 36251 | 1511 | 1124.00 | 8.11 | 0.9893 |
|  | ZW8 | 34076 | 1185 | 763.00 | 4.16 | 0.9878 |
|  | ZW9 | 40854 | 1718 | 1299.00 | 8.49 | 0.9898 |
|  | ZW10 | 39430 | 1517 | 1122.00 | 8.08 | 0.9901 |
|  | ZW11 | 41479 | 1544 | 1154.00 | 8.07 | 0.9907 |
|  | ZW12 | 42170 | 1603 | 1138.67 | 7.83 | 0.9910 |
|  | ZW13 | 37499 | 1568 | 1220.00 | 8.32 | 0.9907 |
|  | ZW14 | 42256 | 1608 | 1248.00 | 8.26 | 0.9917 |
| Duodenum | 12Z4 | 38235 | 1277 | 953.00 | 7.37 | 0.9918 |
|  | 12Z5 | 46067 | 1554 | 996.00 | 7.33 | 0.9915 |
|  | 12Z6 | 36384 | 1422 | 1068.00 | 7.19 | 0.9907 |
|  | 12Z7 | 36208 | 1516 | 1129.00 | 7.59 | 0.9893 |
|  | 12Z8 | 33424 | 1446 | 1085.00 | 7.12 | 0.9892 |
|  | 12Z9 | 38040 | 1166 | 755.00 | 4.58 | 0.9895 |
|  | 12Z10 | 36825 | 1425 | 1080.00 | 8.21 | 0.9906 |
|  | 12Z11 | 36911 | 1346 | 994.00 | 7.57 | 0.9906 |
|  | 12Z12 | 38374 | 729 | 495.00 | 4.96 | 0.9941 |
|  | 12Z13 | 31211 | 784 | 501.00 | 5.3 | 0.9909 |
|  | 12Z14 | 36389 | 996 | 728.00 | 6.54 | 0.9928 |
| Jejunum | KC4 | 37712 | 1053 | 700.50 | 5.87 | 0.9908 |
|  | KC5 | 39545 | 1240 | 799.00 | 5.28 | 0.9891 |
|  | KC6 | 35917 | 766 | 513.00 | 5.42 | 0.9930 |
|  | KC7 | 35125 | 822 | 568.00 | 5.32 | 0.9930 |
|  | KC8 | 37600 | 1011 | 669.50 | 6.38 | 0.9913 |
|  | KC9 | 40823 | 706 | 423.00 | 5.27 | 0.9940 |
|  | KC10 | 36785 | 1357 | 1015.00 | 7.59 | 0.9907 |
|  | KC11 | 37537 | 819 | 531.00 | 5.19 | 0.9925 |
|  | KC12 | 40833 | 767 | 554.00 | 5.82 | 0.9950 |
|  | KC13 | 39642 | 737 | 515.00 | 5.09 | 0.9952 |
|  | KC14 | 35146 | 1171 | 765.00 | 6.76 | 0.9885 |
| Ileum | HC4 | 44799 | 1224 | 758.00 | 5.98 | 0.9919 |
|  | HC5 | 45881 | 1233 | 746.00 | 6.46 | 0.9913 |
|  | HC6 | 37942 | 1237 | 854.00 | 6.49 | 0.9899 |
|  | HC7 | 38076 | 1350 | 969.00 | 6.77 | 0.9897 |
|  | HC8 | 36365 | 1292 | 938.00 | 6.56 | 0.9902 |
|  | HC9 | 40362 | 1280 | 889.00 | 6.25 | 0.9908 |
|  | HC10 | 37413 | 627 | 436.00 | 4.8 | 0.9951 |
|  | HC11 | 41772 | 1272 | 845.50 | 6.1 | 0.9910 |
|  | HC12 | 38908 | 1295 | 934.00 | 6.57 | 0.9908 |
|  | HC13 | 38973 | 1400 | 983.00 | 6.61 | 0.9895 |
|  | HC14 | 40183 | 1346 | 982.00 | 7.15 | 0.9910 |
| Caecum | MC4 | 32006 | 1118 | 737.00 | 5.93 | 0.9881 |
|  | MC5 | 42702 | 1376 | 865.33 | 6.76 | 0.9909 |
|  | MC6 | 38201 | 1355 | 979.00 | 6.96 | 0.9902 |
|  | MC7 | 37590 | 1378 | 952.00 | 6.94 | 0.9887 |
|  | MC8 | 33803 | 1239 | 827.00 | 6.3 | 0.9878 |
|  | MC9 | 36916 | 1304 | 923.00 | 6.44 | 0.9897 |
|  | MC10 | 41168 | 1297 | 962.00 | 6.48 | 0.9916 |
|  | MC11 | 37596 | 1318 | 915.00 | 6.82 | 0.9893 |
|  | MC12 | 41101 | 1340 | 929.25 | 6.75 | 0.9905 |
|  | MC13 | 47093 | 1414 | 897.75 | 6.67 | 0.9921 |
|  | MC14 | 110356 | 1695 | 807.00 | 6.6 | 0.9969 |
| Colon | JC4 | 37067 | 1179 | 826.00 | 6.3 | 0.9905 |
|  | JC5 | 23890 | 1077 | 628.00 | 5.98 | 0.9812 |
|  | JC6 | 36006 | 1313 | 927.00 | 6.89 | 0.9893 |
|  | JC7 | 36139 | 1303 | 886.00 | 6.62 | 0.9885 |
|  | JC8 | 39484 | 1242 | 868.00 | 5.94 | 0.9906 |
|  | JC9 | 41194 | 1357 | 905.00 | 6.44 | 0.9908 |
|  | JC10 | 58576 | 1391 | 816.00 | 5.98 | 0.9941 |
|  | JC11 | 31355 | 1106 | 733.00 | 6.33 | 0.9881 |
|  | JC12 | 40319 | 1357 | 948.33 | 6.82 | 0.9909 |
|  | JC13 | 45504 | 1415 | 848.00 | 6.6 | 0.9918 |
|  | JC14 | 114065 | 1738 | 1005.00 | 7.31 | 0.9974 |
| Faeces | FB4 | 50842 | 1181 | 804.25 | 6.19 | 0.9933 |
|  | FB5 | 47662 | 1167 | 809.00 | 6.49 | 0.9925 |
|  | FB6 | 45281 | 1408 | 821.00 | 6.62 | 0.9918 |
|  | FB7 | 53305 | 1232 | 842.00 | 6.34 | 0.9927 |
|  | FB8 | 61132 | 1143 | 762.00 | 5.89 | 0.9938 |
|  | FB9 | 47867 | 1279 | 928.00 | 6.47 | 0.9927 |
|  | FB10 | 40509 | 1458 | 951.00 | 6.6 | 0.9890 |
|  | FB11 | 35690 | 1245 | 870.00 | 6.25 | 0.9895 |
|  | FB12 | 61251 | 1284 | 883.00 | 6.58 | 0.9935 |
|  | FB13 | 37750 | 1420 | 988.00 | 6.69 | 0.9894 |
|  | FB14 | 51788 | 1767 | 971.20 | 7.30 | 0.9936 |

**Table S2.** The 16S rRNA copy number adjusted counts for phyla present in each sample. See Supplemental_Table_S1.csv.

**Table S3.** Comparison of the predominant phyla (average relative abundance ≥5% in at least one GIT region) in all samples across the gastrointestinal tract within Bactrian camels using the Kruskal-Wallis test. Means in the same row with different superscripts represent a significant difference.

| Phylum | Rumen | Reticulum | Abomasum | Duodenum | Jejunum | Ileum | Caecum | Colon | Faeces | *P*-value |
| --- | --- | --- | --- | --- | --- | --- | --- | --- | --- | --- |
| Firmicutes | 38.41^a^ | 40.90^ab^ | 35.39^a^ | 40.82^a^ | 63.54^b^ | 41.09^a^ | 38.08^a^ | 32.80^a^ | 32.15^a^ | 0.001055 |
| Verrucomicrobia | 11.99^a^ | 9.67^a^ | 10.78^a^ | 8.53^a^ | 3.62^a^ | 34.22^b^ | 34.47^b^ | 36.65^b^ | 37.87^b^ | 1.001e-11 |
| Bacteroidetes | 25.02^ac^ | 26.06^c^ | 25.36^ac^ | 12.74^ab^ | 3.44^b^ | 14.46^abc^ | 17.98^ac^ | 19.99^ac^ | 17.20^ac^ | 8.866e-05 |
| Proteobacteria | 3.79^c^ | 3.70^c^ | 5.82^ac^ | 18.93^ab^ | 18.46^b^ | 4.29^ac^ | 3.16^c^ | 3.90^c^ | 4.69^ac^ | 0.0003955 |
| Actinobacteria | 0.69^cd^ | 0.52^cd^ | 9.53^abd^ | 8.15^ab^ | 6.36^b^ | 0.95^acd^ | 0.63^cd^ | 0.95^c^ | 0.55^cd^ | 3.486e-06 |
| Fibrobacteres | 8.17^b^ | 7.70^b^ | 4.43^b^ | 2.23^ab^ | 0.21^ac^ | 0.02^c^ | 0.02^c^ | 0.04^ac^ | 0.07^ac^ | 2.107e-11 |
| Lentisphaerae | 5.01^d^ | 4.45^cd^ | 3.40^acd^ | 1.55^ab^ | 0.51^b^ | 1.429^ab^ | 1.26^ab^ | 1.65^ac^ | 2.09^acd^ | 1.461e-06 |
| Spirochaetes | 3.23^d^ | 3.43^d^ | 1.92^bd^ | 0.96^a^ | 0.09^c^ | 0.30^ac^ | 0.53^a^ | 0.45^ac^ | 0.89^ab^ | 8.249e-12 |
| Euryarchaeota | 0.20^a^ | 0.03^a^ | 0.04^a^ | 0.13^a^ | 0.02^a^ | 1.82^b^ | 2.60^b^ | 2.47^b^ | 2.92^b^ | 7.367e-11 |
| Planctomycetes | 0.89 | 0.70 | 0.67 | 0.86 | 0.68 | 0.38 | 0.37 | 0.57 | 0.46 | 0.5872 |
| Cyanobacteria | 0.19 | 0.19 | 0.19 | 1.90 | 0.26 | 0.60 | 0.48 | 0.62 | 0.67 | 0.01174 |
| Tenericutes | 0.79^b^ | 0.88^b^ | 1.00^b^ | 0.47^ab^ | 0.59^ab^ | 0.30^a^ | 0.34^a^ | 0.30^a^ | 0.33^a^ | 0.0001834 |
| Synergistetes | 0.62^b^ | 0.72^b^ | 0.59^b^ | 0.36^ab^ | 0.02^ac^ | <0.01^c^ | <0.01^c^ | <0.01^c^ | <0.01^c^ | 4.45e-13 |
| Chloroflexi | 0.30^a^ | 0.33^a^ | 0.26^a^ | 0.34^a^ | 0.52^a^ | 0.03^b^ | 0.01^b^ | 0.01^b^ | 0.01^b^ | 6.181e-12 |
| P_TM7 | 0.21^a^ | 0.18^a^ | 0.12^a^ | 0.22^a^ | 0.75^a^ | 0.04^b^ | 0.02^b^ | 0.02^b^ | 0.01^b^ | 1.272e-10 |
| Acidobacteria | 0.09^a^ | 0.10^a^ | 0.12^a^ | 0.61^a^ | 0.58^a^ | 0.02^b^ | <0.01^b^ | <0.01^b^ | <0.01^b^ | 1.029e-12 |

**Table S4.** The 16S rRNA copy number adjusted counts for genera present in each sample. See Supplemental_Table_S2.csv.

**Table S5.** Comparison of the predominant genera (average relative abundance ≥5% in at least one GIT region) in samples across the gastrointestinal tract of Bactrian camels using the Kruskal-Wallis test. Means in the same row with different superscripts represent a significant difference (*P* < 0.05).

| Taxa | Rumen | Reticulum | Abomasum | Duodenum | Jejunum | Ileum | Caecum | Colon | Faeces | *P-*value |
| --- | --- | --- | --- | --- | --- | --- | --- | --- | --- | --- |
| **Firmicutes** |  |  |  |  |  |  |  |  |  |  |
| *Lactobacillus* | 0.10^bc^ | 0.05^bc^ | 0.17^bc^ | 4.94^ab^ | 6.41^a^ | 0.58^c^ | 0.15^c^ | 0.04^c^ | 0.03^c^ | 4.047e-05 |
| Unclassified Christensenellaceae | 1.85^a^ | 2.10^a^ | 1.90^a^ | 1.94^a^ | 5.32^a^ | 1.03^b^ | 0.68^b^ | 0.59^b^ | 0.77^b^ | 2.027e-07 |
| Unclassified Ruminococcaceae | 6.93^a^ | 7.43^a^ | 6.57^a^ | 6.46^a^ | 5.41^a^ | 14.56^b^ | 15.26^b^ | 14.62^b^ | 13.62^b^ | 8.372e-09 |
| Unclassified Clostridiales | 17.59^b^ | 17.93^b^ | 14.86^ab^ | 14.07^ab^ | 25.78^b^ | 11.69^ac^ | 8.53^ac^ | 6.37^c^ | 7.03^c^ | 9.584e-08 |
| **Verrucomicrobia** |  |  |  |  |  |  |  |  |  |  |
| Unclassified f__RFP12 | 6.92^b^ | 7.41^b^ | 7.09^b^ | 5.92^ab^ | 2.44^a^ | 4.10^ab^ | 3.97^ab^ | 4.49^ab^ | 5.11^ab^ | 0.002102 |
| *Akkermansia* | 2.86^ab^ | 0.02^b^ | 0.15^ab^ | 0.15^ab^ | 0.69^a^ | 29.33^c^ | 29.81^c^ | 31.26^c^ | 31.64^c^ | 1.687e-12 |
| **Proteobacteria** |  |  |  |  |  |  |  |  |  |  |
| *Burkholderia* | 0.17^bc^ | 0.18^c^ | 0.95^c^ | 6.08^ab^ | 6.14^a^ | 0.82^c^ | 0.11^c^ | 0.10^c^ | 0.07^c^ | 1.188e-07 |
| *Pseudomonas* | 0.11^ac^ | 0.09^c^ | 1.05^bc^ | 4.94^a^ | 5.95^a^ | 0.29^bc^ | 0.06^bc^ | 0.03^b^ | 0.05^bc^ | 1.073e-08 |
| **Bacteroidetes** |  |  |  |  |  |  |  |  |  |  |
| Unclassified Bacteroidales | 9.93^c^ | 11.19^c^ | 10.12^c^ | 5.18^a^ | 0.51^b^ | 3.18^ab^ | 4.15^a^ | 4.53^a^ | 4.42^a^ | 5.06e-08 |
| *5-7N15* | 0.13^a^ | <0.01^a^ | 0.02^a^ | 0.01^a^ | 0.12^a^ | 3.77^b^ | 4.22^b^ | 5.51^b^ | 4.71^b^ | 7.628e-12 |
| Unclassified BS11 | 6.82^bd^ | 6.16^d^ | 6.87^bd^ | 2.87^ab^ | 0.59^c^ | 1.24^ac^ | 1.69^ac^ | 2.02^a^ | 1.61^ac^ | 1.452e-07 |
| *Prevotella* | 4.36^b^ | 5.08^b^ | 4.75^b^ | 2.12^ab^ | 0.65^ac^ | 0.07^c^ | 0.06^c^ | 0.07^c^ | 0.08^c^ | 7.655e-11 |
| **Fibrobacteres** |  |  |  |  |  |  |  |  |  |  |
| *Fibrobacter* | 8.17^b^ | 7.70^b^ | 4.43^b^ | 2.23^ab^ | 0.21^ac^ | 0.02^c^ | 0.02^c^ | 0.04^ac^ | 0.07^ac^ | 2.107e-11 |
| **Actinobacteria** |  |  |  |  |  |  |  |  |  |  |
| Unclassified Bifidobacteriaceae | 0.01^de^ | <0.01^e^ | 9.01^bc^ | 7.57^ab^ | 4.13^a^ | 0.39^abc^ | 0.22^abc^ | 0.07^cd^ | 0.07^cd^ | 8.674e-10 |

**Table S6.** Predicted functions of the bacterial microbiota throughout the GIT of Bactrian camels using the Kruskal-Wallis test. Means with the same superscript within the same row are significantly different at *P* < 0.05.

| Functions | Rumen | Reticulum | Abomasum | Duodenum | Jejunum | Ileum | Caecum | Colon | Faeces | *P*-value |
| --- | --- | --- | --- | --- | --- | --- | --- | --- | --- | --- |
| **Cellular Processes** |  |  |  |  |  |  |  |  |  |  |
| Cell Motility | 2.69^bd^ | 2.76^abd^ | 2.68^bde^ | 3.12^ab^ | 3.61^a^ | 2.50^cde^ | 2.30^ce^ | 2.23^c^ | 2.26^c^ | 1.173e-08 |
| Transport and Catabolism | 0.36^ac^ | 0.35^ac^ | 0.36^ac^ | 0.31^ab^ | 0.25^b^ | 0.35^ac^ | 0.37^ac^ | 0.39^c^ | 0.38^c^ | 1.847e-06 |
| Cell Growth and Death | 0.59^b^ | 0.60^b^ | 0.58^b^ | 0.55^ad^ | 0.51^ac^ | 0.50^c^ | 0.50^c^ | 0.50^c^ | 0. 50^c^ | 1.274e-10 |
| Cell Communication | 0.00^c^ | <0.01^abc^ | 0.00^bc^ | <0.01^ab^ | <0.01^b^ | 0.00^c^ | 0.00^c^ | 0.00^c^ | 0.00^c^ | 0.000971 |
| **Environmental Information Processing** |  |  |  |  |  |  |  |  |  |  |
| Membrane Transport | 10.23^c^ | 10.33^c^ | 10.51^ac^ | 11.48^ab^ | 12.93^b^ | 11.25^ab^ | 11.00^ac^ | 11.25^ac^ | 10.76^ac^ | 2.165e-06 |
| Signalling Molecules and Interaction | 0.15^bd^ | 0.14^d^ | 0.16^bd^ | 0.17^ab^ | 0.17^ab^ | 0.20^ac^ | 0.20^ac^ | 0.21^c^ | 0.21^c^ | 4.627e-11 |
| Signal Transduction | 1.81^bcd^ | 1.78^cd^ | 1.76^d^ | 1.97^abc^ | 2.03^a^ | 1.99^a^ | 1.95^ab^ | 1.97^a^ | 2.00^a^ | 2.568e-05 |
| **Genetic Information Processing** |  |  |  |  |  |  |  |  |  |  |
| Folding, Sorting and Degradation | 2.57^c^ | 2.56^bc^ | 2.52^bc^ | 2.40^ab^ | 2.28^a^ | 2.55^c^ | 2.58^c^ | 2.62^c^ | 2.60^c^ | 1.033e-07 |
| Replication and Repair | 9.02^bc^ | 9.13^c^ | 9.12^c^ | 8.44^ab^ | 8.07^a^ | 8.26^a^ | 8.29^a^ | 8.27^a^ | 8.20^a^ | 1.536e-08 |
| Transcription | 2.68^bc^ | 2.68^c^ | 2.65^c^ | 2.71^abc^ | 2.91^d^ | 2.89^d^ | 2.88^d^ | 2.82^abd^ | 2.83^abd^ | 4.021e-07 |
| Translation | 5.96^bc^ | 6.04^c^ | 6.03^c^ | 5.48^ab^ | 5.20^a^ | 5.38^a^ | 5.45^a^ | 5.44^a^ | 5.42^a^ | 3.735e-08 |
| **Human Diseases** |  |  |  |  |  |  |  |  |  |  |
| Cancers | 0.11^a^ | 0.11^a^ | 0.10^a^ | 0.10^a^ | 0.10^a^ | 0.12^b^ | 0.12^b^ | 0.12^b^ | 0.12^b^ | 6.252e-09 |
| Cardiovascular Diseases | <0.01^bc^ | <0.01^bc^ | <0.01^c^ | <0.01^ab^ | <0.01^a^ | <0.01^c^ | <0.01^c^ | <0.01^c^ | <0.01^c^ | 4.372e-08 |
| Immune System Diseases | 0.04^acd^ | 0.04^acd^ | 0.04^ad^ | 0.04^a^ | 0.04^bcd^ | 0.03^b^ | 0.03^b^ | 0.03^bc^ | 0.03^bc^ | 4.303e-06 |
| Infectious Diseases | 0.37^bc^ | 0.37^c^ | 0.37^c^ | 0.39^abc^ | 0.40^abd^ | 0.40^ad^ | 0.40^ad^ | 0.41^d^ | 0.41^d^ | 6.186e-07 |
| Metabolic Diseases | <0.01^bcd^ | <0.01^cd^ | <0.01^d^ | <0.01^abcd^ | <0.01^abc^ | <0.01^ab^ | <0.01^ab^ | <0.01^ab^ | <0.01^a^ | 1.234e-05 |
| Neurodegenerative Diseases | 0.11^c^ | 0.11^c^ | 0.12^bc^ | 0.18^ab^ | 0.19^a^ | 0.13^ab^ | 0.13^ab^ | 0.13^a^ | 0.13^a^ | 3.237e-07 |
| **Metabolism** |  |  |  |  |  |  |  |  |  |  |
| Amino Acid Metabolism | 10.3^ce^ | 10.2^e^ | 10.1^bce^ | 9.99^abc^ | 9.70^d^ | 9.80^ad^ | 9.86^ad^ | 9.90^abd^ | 9.89^abd^ | 1.552e-07 |
| Biosynthesis of Other Secondary Metabolites | 1.00 ^a^ | 1.00^a^ | 1.01^a^ | 0.95^ab^ | 0.89^b^ | 0.98^a^ | 0.99^a^ | 1.01^a^ | 1.01^a^ | 0.000103 |
| Carbohydrate Metabolism | 9.66^ab^ | 9.62^b^ | 9.79^ab^ | 9.81^ab^ | 9.93^ac^ | 10.01^c^ | 10.05^c^ | 10.04^c^ | 10.06^c^ | 2.045e-08 |
| Energy Metabolism | 5.90^c^ | 5.89^c^ | 5.84^c^ | 5.57^ab^ | 5.39^b^ | 5.77^ac^ | 5.87^ac^ | 5.91^c^ | 5.91^c^ | 5.549e-05 |
| Enzyme Families | 2.22^b^ | 2.24^b^ | 2.18^b^ | 2.10^a^ | 2.06^a^ | 2.06^a^ | 2.06^a^ | 2.05^a^ | 2.03^a^ | 3.382e-08 |
| Glycan Biosynthesis and Metabolism | 2.59^bcd^ | 2.53^bd^ | 2.48^bd^ | 2.19^ab^ | 1.83^a^ | 2.65^cd^ | 2.72^cd^ | 2.85^c^ | 2.81^c^ | 2.918e-09 |
| Lipid Metabolism | 3.22 | 3.16 | 3.12 | 3.29 | 3.19 | 3.28 | 3.26 | 3.31 | 3.32 | 0.06905 |
| Metabolism of Cofactors and Vitamins | 4.45 ^de^ | 4.45 ^e^ | 4.38^cde^ | 4.17^ab^ | 3.99 ^b^ | 4.30^abc^ | 4.34^acd^ | 4.37^acde^ | 4.34^acd^ | 2.114e-07 |
| Metabolism of Other Amino Acids | 1.52^a^ | 1.53^a^ | 1.54^a^ | 1.63^a^ | 1.61^a^ | 1.37^b^ | 1.36^b^ | 1.37^b^ | 1.35^b^ | 4.7e-12 |
| Metabolism of Terpenoids and Polyketides | 1.77^ab^ | 1.76^ab^ | 1.75^a^ | 1.77^cd^ | 1.72^bc^ | 1.73^d^ | 1.74^de^ | 1.76^e^ | 1.75^f^ | 0.1911 |
| Nucleotide Metabolism | 4.03^bc^ | 4.08^bc^ | 4.11^c^ | 3.80^ab^ | 3.66^a^ | 3.68^a^ | 3.72^a^ | 3.71^a^ | 3.69^a^ | 8.791e-08 |
| Xenobiotics Biodegradation and Metabolism | 1.74^de^ | 1.71^e^ | 1.89^cde^ | 2.47^ab^ | 2.62^b^ | 1.94^abc^ | 1.88^acd^ | 1.88^acd^ | 1.90^ac^ | 1.693e-08 |
| **Organismal Systems** |  |  |  |  |  |  |  |  |  |  |
| Circulatory System | <0.01^be^ | <0.01^bde^ | <0.01^bde^ | 0.01^ab^ | 0.02^a^ | <0.01^cde^ | <0.01^c^ | <0.01^cd^ | <0.01^cde^ | 2.043e-09 |
| Digestive System | 0.03^ab^ | 0.03^b^ | 0.03^ab^ | 0.02^ab^ | 0.02^ab^ | 0.02^a^ | 0.02^ab^ | 0.02^ab^ | 0.02^a^ | 0.005907 |
| Endocrine System | 0.33^a^ | 0.33^a^ | 0.34^a^ | 0.34^a^ | 0.31^b^ | 0.30^b^ | 0.30^b^ | 0.30^b^ | 0.30^b^ | 6.977e-09 |
| Environmental Adaptation | 0.16^ef^ | 0.16^de^ | 0.16^de^ | 0.15^bc^ | 0.16^ab^ | 0.16^bc^ | 0.16^cd^ | 0.16^cd^ | 0.16^f^ | 0.5081 |
| Excretory System | 0.04^b^ | 0.04^ab^ | 0.04^ab^ | 0.04^ab^ | 0.02^a^ | 0.06^c^ | 0.06^c^ | 0.06^c^ | 0.06^c^ | 2.799e-11 |
| Immune System | 0.09^d^ | 0.09^d^ | 0.08^cd^ | 0.07^ab^ | 0.06^b^ | 0.08^ac^ | 0.08^ac^ | 0.08^ac^ | 0.08^ac^ | 3.95e-08 |
| Nervous System | 0.10^ab^ | 0.10^bc^ | 0.11^ab^ | 0.10^ab^ | 0.10^c^ | 0.10^ab^ | 0.10^ab^ | 0.11^ab^ | 0.11^a^ | 1.771e-05 |
| Sensory System | 0.00^c^ | 0.00^abc^ | 0.00^bc^ | <0.01^ab^ | 0.00^a^ | 0.00^c^ | 0.00^c^ | 0.00^c^ | 0.00^c^ | 0.001024 |
| **Unclassified** |  |  |  |  |  |  |  |  |  |  |
| Cellular Processes and Signalling | 3.95^a^ | 3.90^ef^ | 3.84^a^ | 3.94^a^ | 3.97^a^ | 4.26^b^ | 4.25^b^ | 4.26^b^ | 4.26^b^ | 3.484e-10 |
| Genetic Information Processing | 2.72^a^ | 2.69^a^ | 2.72^a^ | 2.65^a^ | 2.57^a^ | 2.96^b^ | 2.98^b^ | 3.00^b^ | 3.02^b^ | 4.638e-12 |
| Metabolism | 2.51^a^ | 2.46^a^ | 2.51^a^ | 2.57^ab^ | 2.54^a^ | 2.71^c^ | 2.70^bc^ | 2.73^c^ | 2.73^c^ | 8.718e-09 |
| Poorly Characterized | 4.95^a^ | 4.91^a^ | 4.87^a^ | 4.92^a^ | 4.85^a^ | 5.13^b^ | 5.20^b^ | 5.23^b^ | 5.26^b^ | 2.58e-10 |

**Supplementary Figures**

**Fig S1.** Summary of rarefaction results based on operational taxonomic units (OTUs) for each sample. 12Z: duodenum samples; FB: faeces samples; HC: ileum samples; JC: colon samples; KC: jejunum samples; LW: rumen samples; MC: caecum samples; WW: reticulum samples; ZW: abomasum samples.


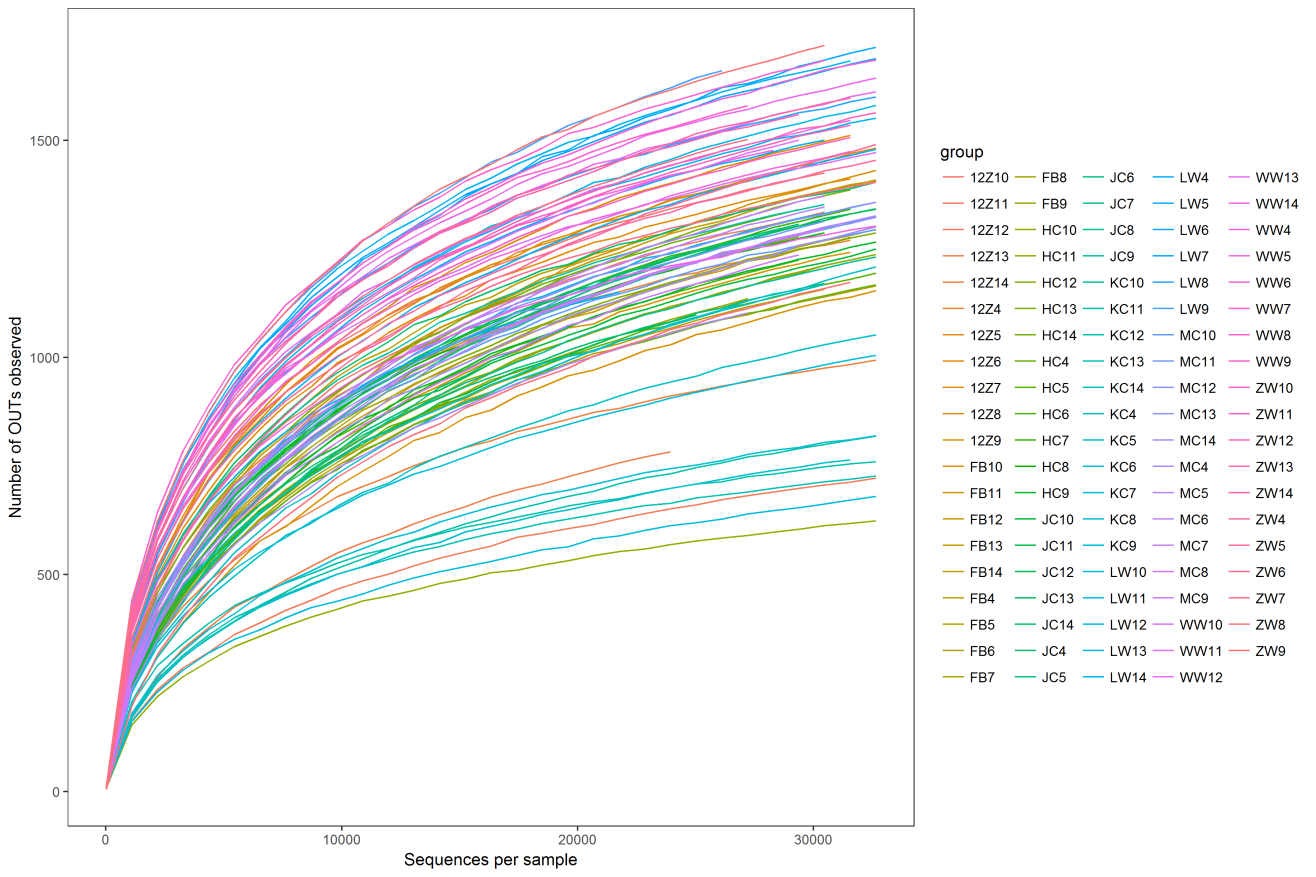


**Fig S2.** Heatmap of hierarchy cluster results for the abundance of genera in different GIT segments. 12Z: duodenum samples; FB: faeces samples; HC: ileum samples; JC: colon samples; KC: jejunum samples; LW: rumen samples; MC: caecum samples; WW: reticulum samples; ZW: abomasum samples.


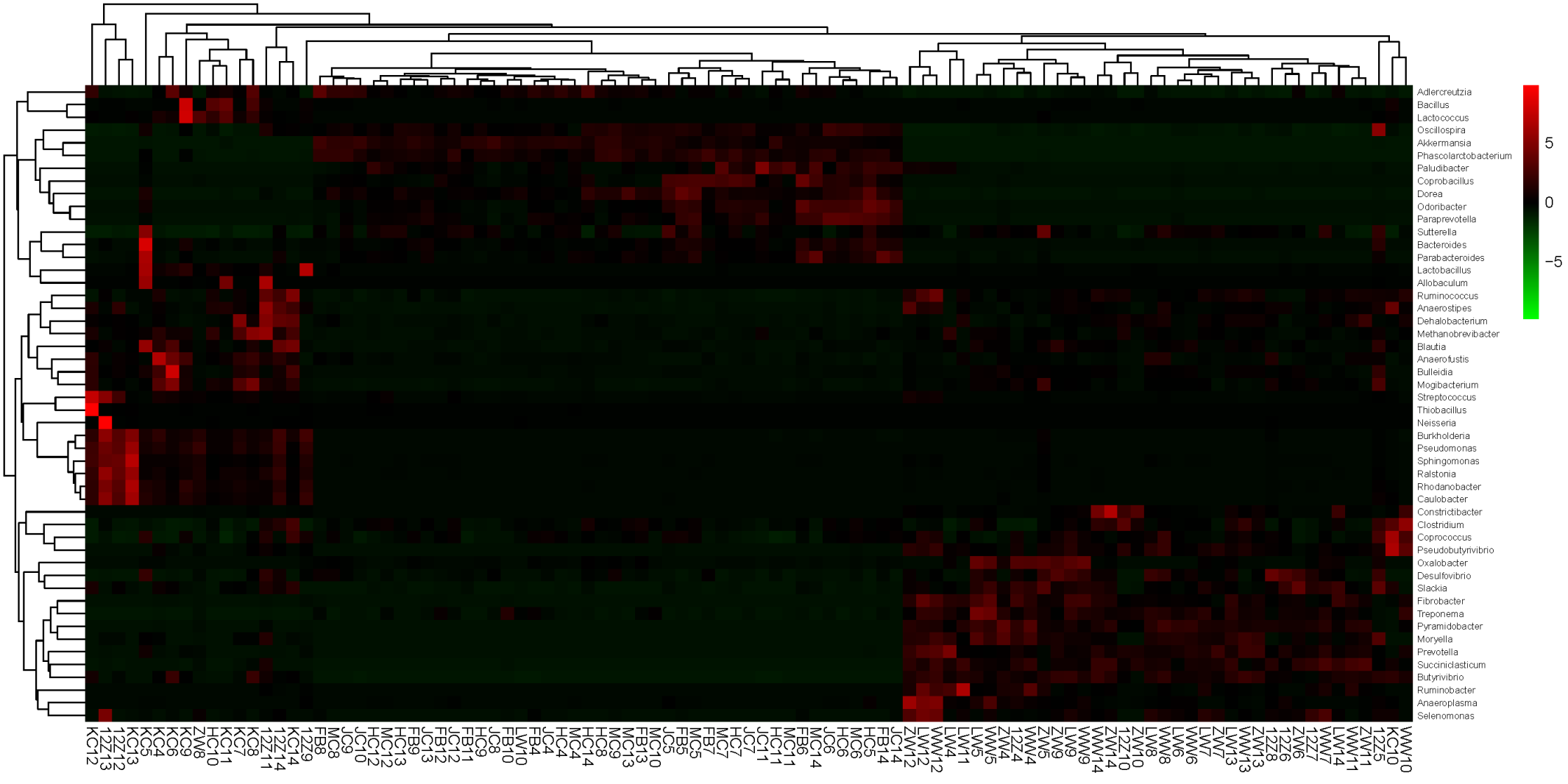

Supplement: Supplementary file 3 — Supplementary information [file 41598_2017_18298_MOESM3_ESM.doc]
